# Long-term effectiveness of Gliadel implant for malignant glioma and prognostic factors for survival: 3-year results of a postmarketing surveillance in Japan

**DOI:** 10.1093/noajnl/vdab189

**Published:** 2022-01-12

**Authors:** Toshihiko Iuchi, Akihiro Inoue, Yuichi Hirose, Motohiro Morioka, Keishi Horiguchi, Atsushi Natsume, Yoshiki Arakawa, Koichi Iwasaki, Minoru Fujiki, Toshihiro Kumabe, Yukinori Sakata

**Affiliations:** 1 Division of Neurological Surgery, Chiba Cancer Center, Chiba, Japan; 2 Department of Neurosurgery, Ehime University School of Medicine, Ehime, Japan; 3 Department of Neurosurgery, Fujita Health University, Aichi, Japan; 4 Department of Neurosurgery, Kurume University School of Medicine, Fukuoka, Japan; 5 Department of Neurosurgery, Gunma University Hospital, Gunma, Japan; 6 Department of Neurosurgery, Nagoya University Hospital, Aichi, Japan; 7 Department of Neurosurgery, Kyoto University Hospital, Kyoto, Japan; 8 Department of Neurosurgery, Tazuke Kofukai Medical Research Institute, Kitano Hospital, Osaka, Japan; 9 Department of Neurosurgery, Oita University Hospital, Oita, Japan; 10 Department of Neurosurgery, Kitasato University Hospital, Kanagawa, Japan; 11 Clinical Planning Department, Medical Headquarters, Eisai Co., Ltd, Tokyo, Japan

**Keywords:** effectiveness, Gliadel, implant, malignant glioma, survival

## Abstract

**Background:**

Adjuvant treatment with Gliadel wafers may prolong overall survival (OS) for malignant glioma patients without increasing toxicity. In Japan, the long-term OS of these patients treated with Gliadel 7.7 mg implants has not been studied. We evaluated OS and prognostic factors that might affect OS in Japanese patients with malignant glioma who received the Gliadel 7.7 mg implant.

**Methods:**

This observational, long-term, postmarketing surveillance was an extension of a previous surveillance. Data were collected through case report forms at 2 and 3 years after Gliadel implant. Up to 8 Gliadel wafers (61.6 mg of carmustine) were placed over the tumor resection site. Primary endpoints were OS and prognostic factors that may influence OS.

**Results:**

Among the 506 patients analyzed, 62.6% had newly diagnosed disease, and 37.4% had recurrent disease; 79.1% had glioblastoma histological type and 79.6% had World Health Organization Grade IV disease. Patients received a median of 8 wafers. The median OS was 18.0 months; OS rates were 39.8% and 31.5% at 2 and 3 years, respectively. Age ≥65 years (hazard ratio [HR]: 1.456; *P* = .002), lower resection rate (HR: 1.206; *P* < .001), recurrence (HR: 2.418; *P* < .001), and concomitant radiotherapy (HR: 0.588; *P* < .001) were identified as significant prognostic factors.

**Conclusions:**

This study confirmed the 2- and 3-year OS of Japanese malignant glioma patients with varied backgrounds after Gliadel implant. With a careful interpretation of indirect comparisons with previously reported data, the results suggest that prognosis could be improved with Gliadel implants.

**Clinical Trial Registration:**

NCT02300506

**Key Points**

- Gliadel wafers plus standard of care yielded survival benefits for malignant glioma.
- Age, resection rate, recurrence, and radiotherapy may predict prognosis.

**Importance of the Study**

To our knowledge, this study is the first to conduct a large-scale analysis of Gliadel use and its effect on the long-term overall survival of Japanese patients with malignant glioma. The present results show that overall survival was prolonged for these patients. Potential prognostic factors determined herein can help identify patients who may benefit the most from therapy with Gliadel wafer implants concomitant with temozolomide and radiotherapy.

Globally, gliomas account for approximately 81% of primary malignant intracranial tumors.^1^ Primary malignant brain and other central nervous system (CNS) tumors have an average annual incidence of 7.08 cases/100 000 persons.^2^ Collectively, these tumors are referred to as malignant gliomas and are graded based on their malignant behavior according to the World Health Organization (WHO) Grades I–IV.^3^ Reportedly, the median survival of patients with high-grade malignant glioma (glioblastoma [GB] and anaplastic astrocytoma [AA]) is 8–18 months.^2^ GB is the most common and most malignant glioma subtype (45% of all malignant gliomas) and has a relative 5-year survival rate of approximately 5%–16%.^1,4^

The most common initial approach and the standard of care for malignant gliomas is maximal safe surgery, followed by radiotherapy, and in some cases, chemotherapy.^5^ However, efficient delivery of drugs to the brain and the CNS is challenging; thus, new drug delivery methods are needed. Carmustine (1,3-bis[2-chloroethyl]-1-nitrosourea) is a nitrosourea alkylating agent that exerts its antitumor effect by alkylating DNA and RNA. The carmustine wafer implant (Gliadel 7.7 mg implant, referred to hereafter as Gliadel) is a biodegradable copolymer (prolifeprospan 20) impregnated with carmustine. This method allows for the controlled-release delivery of carmustine within the confines of the brain tumor environment.^6–9^

A phase 3 study conducted in 14 countries, including the United States, Germany, France, the United Kingdom, Scotland, Finland, and Israel, suggested a prolongation of overall survival (OS) in newly diagnosed patients with malignant glioma who received Gliadel wafer implants.^10^ The median OS was 13.9 months for the Gliadel wafer-treated group and 11.6 months for the placebo-treated group (log-rank *P* = .03 stratified by country), with a 29% reduction in the risk of death in the treatment group.^10^ The results of a recently conducted retrospective study^11^ and a systematic review^12^ suggested adjuvant radiotherapy with concomitant and adjuvant temozolomide (so-called Stupp protocol), and Gliadel wafers may contribute to improving OS without increasing toxicity. In patients with recurrent malignant gliomas, the median OS of the 110 patients who received carmustine polymers was 31 weeks compared with 23 weeks for the 112 patients who received only placebo polymers (hazard ratio [HR]: 0.67, *P* = .006).^13^ More recently, a phase 1/2 clinical trial was conducted in Japan to evaluate the efficacy, safety, and pharmacokinetics of Gliadel implants in 16 patients with newly diagnosed malignant gliomas and 8 patients with recurrent malignant gliomas. Patients with newly diagnosed malignant gliomas achieved OS rates of 100.0% and 68.8% at 12 and 24 months, respectively.^14^

Based on the available clinical evidence, the marketing approval for Gliadel as adjuvant therapy for malignant glioma was obtained in September 2012 in Japan, and the implant was marketed from January 2013. Thus far, data on the long-term OS of patients who received Gliadel wafers as adjuvant therapy for malignant glioma in Japan are limited. Therefore, we conducted a postmarketing surveillance to investigate the long-term OS of malignant glioma patients with Gliadel wafer implants in Japan. Previously, we described the safety results of 558 patients who received Gliadel wafers as adjuvant therapy for malignant glioma after the market launch of Gliadel on January 9, 2013 to July 10, 2013.^15^ However, the evaluation period included in our previous surveillance was too short to evaluate the long-term OS in patients with Gliadel wafer implants. Therefore, we conducted a postmarketing surveillance of long-term observation to investigate the prognosis of patients with malignant glioma and prognostic factors of these patients based on extended observation of patients who were surveyed and whose case report forms (CRFs) were collected in our previous surveillance.

## Materials and Methods

### Study Design and Eligibility

A postmarketing surveillance was previously conducted by Eisai Co., Ltd, which examined all patients with malignant glioma who received treatment with Gliadel since its launch in accordance with the principles of Good Post-Marketing Study Practice (GPSP) in Japan.^15^ This study was an extension of the previous surveillance, and it was conducted between April 1, 2014 and March 31, 2017. The total observation period after Gliadel wafer placement was 3 years.

GPSP, an authorized guideline for postmarketing surveillance, does not require the establishment of an institutional review board or ethics committee. However, the data in this surveillance were collected with due consideration of the Declaration of Helsinki and data integrity, in compliance with the personal information protection law in Japan. Patients who participated in the previous surveillance, agreed to participate in the current study, and provided signed informed consent were enrolled using a central registration system from the previous surveillance sites.

CRFs were collected at the end of the second and third years after receiving the Gliadel implant. The data from the CRFs were recorded by attending physicians. According to the size and shape of the tumor resection cavity, up to 8 wafers of Gliadel, impregnated with 61.6 mg of carmustine, were used to cover the brain tumor resection site.

### Effectiveness

The endpoints were OS and prognostic factors (patient background factors at baseline and factors after Gliadel implant) that may be associated with OS outcomes. The OS was defined as the period from the day of Gliadel implant placement until death. For patients who survived past the final observation, the OS period was censored by the day of the final confirmation of survival. For these analyses, OS results were stratified by patient background characteristics, and OS was the responding variable. Analyses of OS and prognostic factors were performed in all patients and patients with newly diagnosed or recurrent glioma.

In Japanese real-world clinical practice, concurrent temozolomide and radiation are usually performed as standard of care after surgery; the Stupp protocol is listed as a Grade A recommendation in the Japanese guidelines.^16^ The present study investigated prognosis after Gliadel implantation in clinical practice; therefore, a prospective evaluation using the strictly defined Stupp protocol was not conducted. Instead, we performed an exploratory subgroup analysis of the long-term prognosis in subgroups under similar conditions to those in the Stupp protocol. This analysis included patients ≤70 years/>70 years of age with newly diagnosed GB who had received concurrent temozolomide and concurrent radiotherapy. A separate analysis was also conducted that included patients ≤70 years of age with newly diagnosed GB, a resection rate ≥95%, and who had received concurrent temozolomide and radiotherapy.

### Statistical Analysis

The planned sample size of this study was up to 560 Gliadel recipients as this was the number of patients whose CRFs were collected in the previous surveillance when this study was planned. For patient disposition, data were obtained regarding the number of patients for whom a CRF was obtained.

Patient background data were summarized using distributions for categorical data and descriptive statistics (mean, standard deviation, median, and minimum and maximum) for quantitative data. The OS was summarized using descriptive statistics (number of patients, quartile points [25%, median, 75%], minimum, and maximum). The rate of events (deaths) among censored subjects was calculated. The survival rates at each time point with 95% confidence intervals (CIs) were calculated by the Kaplan–Meier method, and Kaplan–Meier curves were plotted to show OS rate at each time point. The same analyses were performed using populations stratified by resection rate (<95%, ≥95%), age (non-elderly, <65 years; elderly, ≥65 years), and WHO classification (Grade III, Grade IV). These stratification factors were applied to both the newly diagnosed and recurrent subgroups. Prognostic factors that may be associated with OS outcomes were investigated using a Cox proportional hazards model. More specifically, Cox regression analyses (univariate analysis, multivariate analysis [full model], and multivariate analysis [selection according to the step-down method]) were conducted for the following patient demographic factors to identify the factors that were associated with OS outcomes: sex, age (<65 years, ≥65 years), weight (continuous variable), history of allergy, past medical history, complications (cerebral edema, renal dysfunction, hepatic dysfunction, or other complications), primary or recurrent glioma, resection rate (100, ≥95 to <100, ≥90 to <95, ≥80 to <90, or <80), histopathological diagnosis, number of tumors, number of implanted wafers, Karnofsky Performance Status immediately before implantation of this drug (10–70 vs 80–100), concomitant anticancer agents for malignant glioma, concomitant radiotherapy for malignant glioma, use of a fixation agent for Gliadel, recurrence of glioma after implantation of Gliadel, and elimination of remnant by 30 days after Gliadel implantation. All analyses were performed using 2 populations (stratified by newly diagnosed and recurrent Grade IV glioma). The significance level was set at 5%. All statistical analyses were conducted using SAS System Release 9.3 (SAS Institute Japan Ltd).

## Results

### Patient Disposition, Background Characteristics, and Gliadel Placement

Of the 561 patients enrolled in this study (all patients who received treatment with Gliadel since its launch), 507 patients at 203 sites were enrolled between April 1, 2014 and March 31, 2017. One patient was excluded from the OS analysis for being counted twice.

Patient background characteristics are given in Table 1. There were more male than female patients (58.3% and 41.3%, respectively), and the median age was 63 (range: 18–92) years. Among all enrolled patients, 62.6% had a newly diagnosed disease, and 37.4% had recurrent disease; 79.1% had GB histological type, and 79.6% had WHO Grade IV disease. Of the 403 patients with WHO Grade IV disease, 3 patients had gliosarcoma, and the other 400 patients had GB.

**Table 1.** Background Characteristics of Patients

Characteristic		N = 506
Sex	Male	295 (58.3)
	Female	209 (41.3)
	Unknown	2 (0.4)
Age (years)	Median (minimum–maximum)	63 (18–92)
	15 to <65	285 (56.3)
	≥65	221 (43.7)
Past medical history	No	342 (67.6)
	Yes	159 (31.4)
	Unknown/not specified	5 (1.0)
Newly diagnosed glioma or recurrent disease	Newly diagnosed	317 (62.6)
	Recurrent	189 (37.4)
	First recurrence	133 (26.3)
	Second recurrence	33 (6.5)
	Third recurrence	14 (2.8)
	Fourth recurrence	9 (1.8)
Resection rate (%)	100	132 (26.1)
	≥95 to <100	165 (32.6)
	≥90 to <95	69 (13.6)
	≥80 to <90	50 (9.9)
	<80	89 (17.6)
	Unknown or missing	1 (0.2)
	Mean ± standard deviation	87.0 ± 18.8
	Minimum–maximum	5–100
Histopathological type	Glioblastoma	400 (79.1)
	Anaplastic astrocytoma	39 (7.7)
	Anaplastic oligodendroglioma	33 (6.5)
	Anaplastic oligoastrocytoma	13 (2.6)
	Anaplastic ependymoma	6 (1.2)
	Other malignant gliomas	15 (3.0)
WHO Grade	IV	403 (79.6)
	III	103 (20.4)
	II	0 (0.0)
Karnofsky Performance Status	Mean ± standard deviation	71.3 ± 21.3
	≥80 to ≤100	256 (50.6)
	≥10 to ≤70	250 (49.4)
Chromosome 1p/19q codeletion	Not assayed	406 (80.2)
	Assayed^a^	100 (19.8)
	Details	
	Codeletion negative	67 (13.2)
	Codeletion positive	16 (3.2)
	Unknown or missing	0 (0)
MGMT gene methylation	Not assayed	357 (70.6)
	Assayed^a^	149 (29.4)
	Details	
	Positive	69 (13.6)
	Negative	60 (11.9)
	Unknown or missing	0 (0)
Number of Gliadel wafers placed	1	4 (0.8)
	2	15 (3.0)
	3	20 (4.0)
	4	25 (4.9)
	5	29 (5.7)
	6	41 (8.1)
	7	28 (5.5)
	8	344 (68.0)
	Median (range)	8 (1–8)
Any concomitant drug therapy	No	42 (8.3)
	Yes	462 (91.3)
	Unknown or missing	2 (0.4)
Concomitant drugs	Temozolomide	433 (85.6)
	Bevacizumab	182 (36.0)
	Interferon β	46 (9.1)
	ACNU monotherapy	12 (2.4)
	ICE regimen	8 (1.6)
	PAV regimen	5 (1.0)
	Other	21 (4.2)
Concomitant combination with radiotherapy	None	153 (30.2)
	Yes	350 (69.2)
	Unknown or missing	3 (0.6)

ACNU, nimustine; ICE regimen, ifosfamide, carboplatin, and etoposide; PAV regimen, procarbazine, nimustine, and vincristine; WHO, World Health Organization. Data in the table are n (%), unless otherwise indicated.

^a^Including the patients whose data were not provided by the study site.

The majority of patients (68.0% [344/506]) received 8 Gliadel wafers. The median number of wafers placed was 8 wafers. The percentage of patients who underwent a second placement of Gliadel was 6.9% (35/506).

Concomitant therapies after Gliadel placement are also given in Table 1. Most patients (91.3%) received concomitant drug therapy after Gliadel placement. The most common concomitant therapy was temozolomide, used in 85.6% of patients; bevacizumab was used in 36.0% of patients, and 69.2% received radiotherapy.

### OS in All Patients

**Figure 1.**
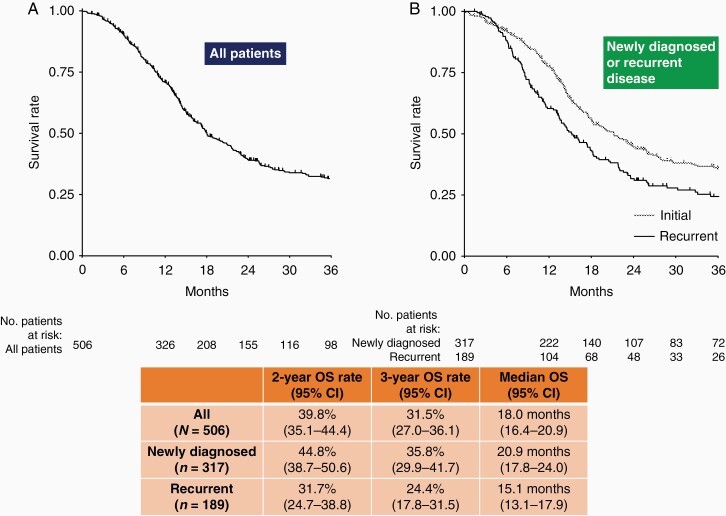
Median overall survival and overall survival rate at 2 and 3 years in all patients (A) and stratified by newly diagnosed or recurrent disease (B). OS, overall survival; CI, confidence interval.

The median OS for all patients was 18.0 months (Figure 1). The OS rate for all patients was 39.8% and 31.5% at 2 and 3 years, respectively, after Gliadel implant placement. When stratified by newly diagnosed and recurrent malignant glioma, the median OS was 20.9 months for patients with newly diagnosed glioma and 15.1 months for patients with recurrent glioma. The OS rate at 2 years was 44.8% and 31.7%, respectively, and the OS rate at 3 years was 35.8% and 24.4%, respectively.

### OS in Patients With Newly Diagnosed Glioma

OS in patients with newly diagnosed glioma according to subgroup is given in Table 2. When stratified by resection rate, the median OS was 24.0 months for patients with a resection rate ≥95% (182/317 patients) and 16.5 months for patients with a resection rate <95% (135/317). The OS rate was 50.2% and 36.9%, respectively, at 2 years and 40.2% and 29.4%, respectively, at 3 years.

**Table 2.** Overall Survival According to Subgroup

	Median (months) (95% CI)	2-Year OS (%) (95% CI)	3-Year OS (%) (95% CI)
Newly diagnosed glioma (n = 317)			
Resection rate ≥95% (n = 182)	24.0 (20.3–32.2)	50.2 (42.3–57.7)	40.2 (32.4–47.9)
Resection rate <95% (n = 135)	16.5 (14.0–20.8)	36.9 (27.7–46.1)	29.4 (20.6–38.7)
Age <65 years (n = 149)	25.6 (20.8–NE)	53.2 (44.6–61.1)	44.8 (36.4–52.9)
Age ≥65 years (n = 168)	16.5 (14.6–20.9)	35.8 (27.4–44.3)	25.0 (17.1–33.7)
WHO Grade III (n = 51)	NR (32.2–NE)	72.8 (56.9–83.6)	64.7 (48.1–77.2)
WHO Grade IV (n = 266)	18.0 (16.0–21.7)	39.4(33.0–45.8)	30.2 (24.1–36.5)
Recurrent glioma (n = 189)			
Resection rate ≥95% (n = 115)	17.9 (14.3–21.7)	36.2 (27.0–45.5)	28.8 (20.0–38.1)
Resection rate <95% (n = 73)	10.9 (8.6–14.4)	24.5 (14.7–35.7)	17.2 (8.4–28.6)
Age <65 years (n = 136)	15.9 (13.3–21.5)	34.4 (26.2–42.7)	27.0 (19.2–35.4)
Age ≥65 years (n = 53)	13.2 (9.3–16.1)	23.5 (11.6–37.9)	16.8 (6.7–30.9)
WHO Grade III (n = 52)	32.3 (21.5–NE)	58.7 (43.4–71.2)	45.0 (29.6–59.2)
WHO Grade IV (n = 137)	13.4 (10.9–15.1)	21.2 (14.3–29.0)	16.5 (10.1–24.2)

CI, confidence interval; NR, not reached; NE, not estimated; OS, overall survival; WHO, World Health Organization.

When stratified by age (<65 and ≥65 years), the median OS was 25.6 months for non-elderly patients (<65 years; 149/317 patients) and 16.5 months for elderly patients (≥65 years; 168/317). The OS rate was 53.2% and 35.8%, respectively, at 2 years and 44.8% and 25.0%, respectively, at 3 years.

When stratified by WHO Grade, the median OS was not reached (95% CI: 32.2–NE) for patients with Grade III WHO classification (51/317 patients) and 18.0 months for patients with Grade IV WHO classification (266/317). The OS rate was 72.8% and 39.4%, respectively, at 2 years and 64.7% and 30.2%, respectively, at 3 years.

When stratified by type (exploratory), the OS rate at 2 years in patients with GB (264/317 patients), AA (18/317), and anaplastic oligodendroglioma/anaplastic oligoastrocytoma (AO/AOA; 24/317) was 39.8% (95% CI: 33.3–46.2), 48.3% (95% CI: 22.5–70.1), and 87.1% (95% CI: 65.0–95.7) and that at 3 years was 30.5% (95% CI: 24.3–36.9), 48.3% (95% CI: 22.5–70.1), and 76.2% (95% CI: 51.5–89.5), respectively.

**Figure 2.**
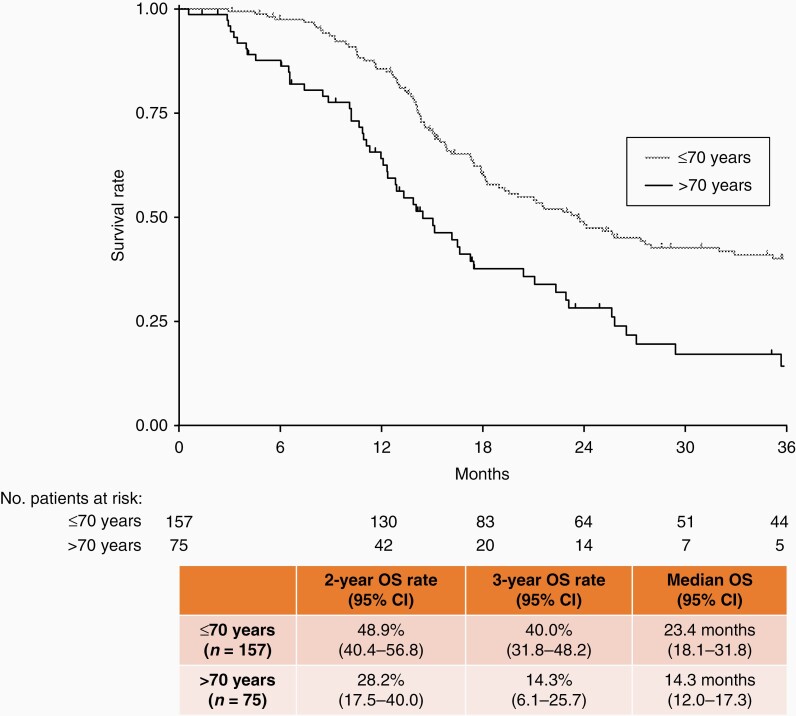
Median overall survival and overall survival rate at 2 and 3 years in patients concomitantly treated with temozolomide and radiation, stratified by age (≤70 years or >70 years). OS, overall survival; CI, confidence interval.

**Figure 3.**
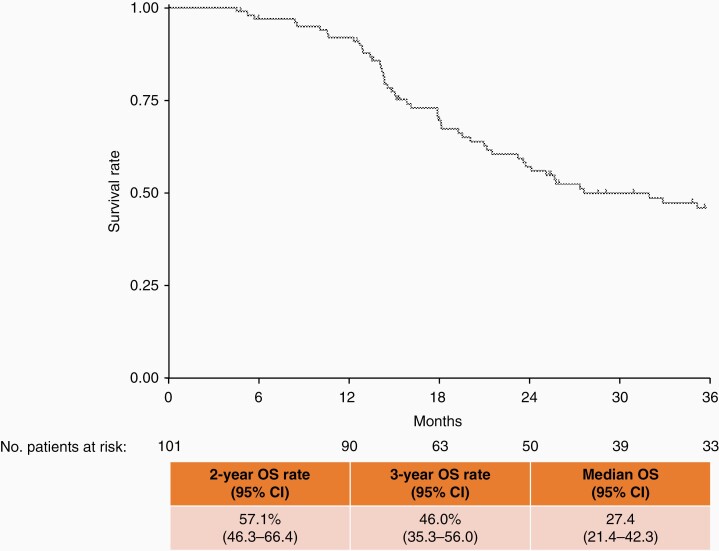
Survival curve of patients aged ≤70 years with newly diagnosed glioblastoma, a resection rate ≥95%, and concomitant therapy with temozolomide and radiation. OS, overall survival; CI, confidence interval.

An exploratory subgroup analysis to evaluate prognosis after Gliadel implant was performed in patients who had received concomitant temozolomide and radiotherapy, which is the standard of care after surgery in clinical practice in Japan. When these patients were extracted and stratified by age (≤70 and >70 years), those aged ≤70 years had a median OS of 23.4 months and a survival rate of 48.9% at 2 years and 40.0% at 3 years (Figure 2). Further exploratory subgroup analyses revealed that patients aged ≤70 years, with a resection rate ≥95%, and who had received concomitant temozolomide and radiotherapy had a median OS of 27.4 months and a survival rate of 57.1% at 2 years and 46.0% at 3 years (Figure 3).

### OS in Patients With Recurrent Glioma

OS in patients with recurrent glioma according to subgroup is given in Table 2. When stratified by resection rate, the median OS was 17.9 months for patients with a resection rate ≥95% (115/189 patients) and 10.9 months for patients with a resection rate <95% (73/189). The OS rate was 36.2% and 24.5%, respectively, at 2 years and 28.8% and 17.2%, respectively, at 3 years.

When stratified by age (<65 and ≥65 years), the median OS was 15.9 months for 136/189 patients <65 years and 13.2 months for 53/189 patients ≥65 years. The OS rate was 34.4% and 23.5%, respectively, at 2 years and 27.0% and 16.8%, respectively, at 3 years.

When stratified by WHO Grade, the median OS was 32.3 months for patients with Grade III gliomas (52/189 patients) and 13.4 months for patients with Grade IV gliomas (137/189). The OS rate was 58.7% and 21.2%, respectively, at 2 years and 45.0% and 16.5%, respectively, at 3 years.

When stratified by type (exploratory), the median OS was 13.4 months (95% CI: 11.1–15.1) for patients with GB (136/189 patients), 25.5 months (95% CI: 8.0–NE) for patients with AA (21/189), and not reached (95% CI: 21.5–NE) for patients with AO/AOA (22/189). The OS rate was 21.4% (95% CI: 14.4–29.2), 53.8% (95% CI: 29.9–72.8), and 73.9% (95% CI: 47.8–88.3), respectively, at 2 years and 16.6% (95% CI: 10.2–24.4), 41.5% (95% CI: 19.3–62.6), and 53.3% (95% CI: 27.0–74.0), respectively, at 3 years.

### Prognostic Factors That Could Influence OS

Prognostic factors evaluated for association with OS outcomes in all patients who received Gliadel implants are given in Table 3. Factors that were significantly associated with OS outcomes after multivariate analysis (step-down method) were age ≥65 years (HR: 1.456, *P* = .002); lower resection rate (HR: 1.206, *P* < .001); histopathological diagnosis of AA (HR: 0.503, *P* = .007), AO (HR: 0.224, *P* < .001), and AOA (HR: 0.264, *P* = .022); number of Gliadel wafers used (HR: 1.115, *P* = .007); concomitant radiotherapy (HR: 0.588, *P* < .001); and recurrence (HR: 2.418, *P* < .001).

**Table 3.** Factors That Could Influence OS in All Patients With Gliadel Implants

Background	Criterion Variable	Evaluation Variable	Univariate Analysis		Multivariate Analysis (Full model)		Multivariate Analysis (Step-downmethod)	
			Hazard Ratio	*P*	Hazard Ratio	*P*	Hazard Ratio	*P*
Sex	Female	Male	1.224	.084	1.369	.033		
Age (years)	<65	≥65	1.490	<.001	1.421	.010	1.456	.002
Weight	Per 1-kg increment		0.992	.105	0.988	.065		
History of allergy	No	Yes	1.064	.727	1.422	.064		
Past medical history	No	Yes	1.063	.625	0.921	.559		
Concomitant disease (cerebral edema)	No	Yes	1.378	.008	1.256	.087		
Complications (renal dysfunction)	No	Yes	0.842	.807	0.454	.437		
Complications (hepatic dysfunction)	No	Yes	1.221	.658	1.352	.516		
Complications (other)	No	Yes	1.117	.343	1.194	.166		
Newly diagnosed glioma or recurrent disease	Newly diagnosed glioma	Recurrent	1.424	.002	1.254	.209		
Resection rate (per 1 category decrement) (100%, ≥95 to <100%, ≥90 to <95%, ≥80 to <90, <80)			1.202	<.001	1.210	<.001	1.206	<.001
Anaplastic astrocytoma (AA)	GB	AA	0.588	.022	0.499	.007	0.503	.007
Anaplastic oligodendroglioma (AO)	GB	AO	0.301	<.001	0.254	<.001	0.224	<.001
Anaplastic oligoastrocytoma (AOA)	GB	AOA	0.205	.006	0.250	.019	0.264	.022
Single or multiple gliomas	Single	Multiple	1.466	.015	1.268	.171		
Number of Gliadel implants	Per 1 implant increment		1.088	.020	1.120	.008	1.115	.007
Karnofsky Performance Status before placement of Gliadel	80–100	10–70	1.655	<.001	1.028	.839		
Concomitant antitumor agent	No	Yes	0.535	.003	0.750	.279		
Concomitant radiotherapy	No	Yes	0.570	<.001	0.732	.102	0.588	<.001
Recurrent after placement	No	Yes	2.326	<.001	2.507	<.001	2.418	<.001
Fixation agent for Gliadel	No	Yes	0.784	.228	0.735	.172		
Elimination of remnant by 30 days after Gliadel	No	Yes	1.088	.612	0.930	.699		

GB, glioblastoma; OS, overall survival.

For patients with newly diagnosed Grade IV glioma, factors that were significantly associated with OS outcome after multivariate analysis (with selection by the backward elimination method) were age ≥65 years (HR: 1.769, *P* = .001), past medical history (HR: 0.622, *P* = .010), lower resection rate (HR: 1.238, *P* < .001), concomitant chemotherapy (HR: 0.154, *P* < .001), and recurrence after Gliadel placement (HR: 1.803, *P* = .001).

For patients with recurrent Grade IV glioma, factors that were significantly associated with OS outcome after multivariate analysis (with selection by the backward elimination method) were lower resection rate (HR: 1.288, *P* < .001) and recurrent disease (HR: 2.828, *P* = .002).

## Discussion

This study analyzed the consecutive drug use surveillance data of 506 patients with malignant glioma who underwent implantation with Gliadel wafers and focused on the long-term OS and prognostic factors associated with OS in these patients. Thus far, this is the first study to analyze the long-term survival outcomes in a large population of patients treated with Gliadel wafers in Japan. The present noninterventional observational study was not placebo-controlled. In a real-world setting, Japanese patients with newly diagnosed and recurrent gliomas who received Gliadel implant achieved a median OS of 20.9 months and 15.1 months, respectively. The 2-year OS rate was 44.8% in newly diagnosed patients and 31.7% in recurrent patients, while the 3-year OS rate was 35.8% and 24.4%, respectively.

Previous international studies reported that implantation with Gliadel wafers provided relevant survival benefits (median OS of approximately 13–14 months) to patients with newly diagnosed malignant glioma.^10,17^ Survival benefits have also been reported for patients with recurrent gliomas. However, the median OS was lower (31 weeks [approximately 7.1 months])^13^ than that reported for patients with newly diagnosed gliomas. A recent study in Japan reported that the OS rate was 68.8% at 24 months in patients with newly diagnosed gliomas and 25% in those with recurrent glioma.^14^ Our results suggest a beneficial effect of Gliadel wafers (perioperative) when administered concurrently with the standard of care on the survival of patients with malignant gliomas in real-world clinical practice.

In patients who received adjuvant treatment with Gliadel for malignant glioma in this study, a subgroup analysis revealed that a good prognosis was observed in newly diagnosed patients with a high resection rate (>95%), aged <65 years, and WHO Grade III tumors. Additionally, a Japanese registry study of patients with malignant glioma showed a relatively high survival rate in patients with a high resection rate and lower WHO Grade tumors.^4^ When considering those findings, we think that our study confirmed a good postoperative prognosis for malignant glioma in a similar patient population. We also conducted an exploratory subgroup analysis of patients in our study who had similar background characteristics to patients in the EORTC–NCIC trial. In this trial,^18^ adult patients aged 18–70 years with newly diagnosed GB who received standard of care with temozolomide and postoperative radiotherapy (the Stupp protocol) showed an OS rate of 27.2% (95% CI: 22.2–32.5) and 16.0% (95% CI: 12.0–20.6) at 2 and 3 years, respectively. Patients with complete resection showed OS rates of 38.4% (95% CI: 29.4–47.3) and 21.4% (95% CI: 14.3–29.6) at 2 and 3 years, respectively. Patients included in our exploratory analysis had newly diagnosed GB, had received concomitant temozolomide and radiotherapy, and were ≤70 years of age. An additional subgroup analysis of these patients according to resection rate (≥95%) was also conducted. We note that these exploratory subgroup analyses revealed that 2- and 3-year OS rates were 48.9% (95% CI: 40.4–56.8) and 40.0% (95% CI: 31.8–48.2), respectively, and were numerically more favorable in patients with newly diagnosed GB who received concomitant temozolomide and radiotherapy; the same could also be said when patients were further stratified by tumor resection rate (≥95%) (2- and 3-year OS rates were 57.1% [95% CI: 46.3–66.4] and 46.0% [95% CI: 35.3–56.0], respectively). As this is not a direct comparison and the treatment environment between a clinical trial and postmarket clinical practice is quite different, careful interpretation is required. In the recurrent GB subgroup of this study, the median OS was 13.4 months and the 2- and 3-year OS rates were 21.4% and 16.6%, respectively. Previously conducted international clinical trials investigating postoperative Gliadel placement for patients with recurrent GB have reported a median OS of 35.3–50.3 weeks (8.1–11.6 months).^19,20^ Our findings, which are comparable to those previously reported, suggest that postoperative Gliadel placement may improve the prognosis in Japanese patients with recurrent GB, which is generally considered to have a poor prognosis.^1,4^

In patients who received adjuvant treatment with Gliadel for malignant glioma in the present study, multivariate analysis (step-down method) suggested that several variables were determinants of OS in all patients. Variables that indicated a good prognosis were histological subtype (AA, HR: 0.503, *P* = .007; AO, HR: 0.224, *P* *<* .001; AOA, HR: 0.264, *P* = .022) and concomitant radiotherapy (HR: 0.588, *P* < .001). We speculate that survival was longer in patients with less aggressive tumors compared with those with more aggressive Grade IV gliomas, thus explaining the association with histological subtype. Regarding concomitant radiotherapy, it was considered that survival benefit might be prolonged due to the therapeutic benefit of this treatment.

Variables that indicated a poor prognosis were age ≥65 years (HR: 1.456, *P* = .002), lower resection rate (per 1 category decrement; HR: 1.206, *P* < .001), number of Gliadel wafers implanted (per 1 implant increment; HR: 1.115, *P* = .007), and recurrence of glioma after Gliadel wafer implantation (HR: 2.418, *P* < .001). In the case of age, we considered that the general decline in the physical health of elderly patients was associated with shorter survival. We also considered that a higher resection rate indicated greater effectiveness of surgery, which would likely prolong survival. Importantly, resection rate was identified as a significant factor affecting OS in all 3 analyses (all patients, newly diagnosed, and recurrent Grade IV glioma). The trend of a prolonged OS with increased resection rate in patients with newly diagnosed GB has been reported previously.^21^ More wafers may have been implanted in patients with high tumor burden and wider tumor resection areas, suggesting that the number of Gliadel wafers was a significant prognostic factor affecting OS. This is a tentative hypothesis as preoperative tumor burden was not investigated. Regarding recurrence, progression of the primary disease was presumably more rapid in patients with recurrence compared with those without, leading to an association between recurrence and a shorter survival time.

Our study revealed that resection rate, age, histological type, and WHO Grade are important factors associated with OS after implantation of Gliadel wafers. Improvement of prognosis in Japanese patients with malignant glioma by implanting Gliadel wafers was indicated. When considering factors that affect OS with Gliadel treatment, resection rate (prior to implantation) and tumor mass were suggested to be important predictors of long-term prognosis.

This study had several limitations, such as those inherent to drug use surveys, including the heterogeneity of treatment of patients with recurrent disease and lack of randomization and comparator groups. The results have limited generalizability to other ethnic populations.

In conclusion, we confirmed the 2- and 3-year OS of Japanese patients with malignant glioma after Gliadel implant (7.7 mg) in the clinical practice setting. Until now, long-term data in Japanese patients treated with Gliadel implants have been limited. Although this study included patients with varied backgrounds, the results suggest that prognosis could be improved with a Gliadel implant.

## Data Availability

The detailed data that support the findings of this study are not available due to the sponsoring company’s policy, except upon reasonable request.

## Notes

**Conflict of interest statement**. In reference to the ICMJE disclosure of interest (http://www.icmje.org/disclosure-of-interest/), we disclose the following conflicts of interest. The study sponsor, Eisai Co., Ltd, participated in the study design; data collection, analysis, and interpretation; writing of the report; and decision to submit the paper for publication. Y.H.: research grants (Eisai Co., Ltd, Otsuka Pharmaceutical Co., Ltd, Takeda Pharmaceutical Company Limited., Daiichi Sankyo Company, Limited, Nippon Boehringer Ingelheim Co., Ltd, Mochida Pharmaceutical Co., Ltd, Chugai Pharmaceutical Co., Ltd, Mitsubishi Tanabe Pharma Corporation., Pfizer Japan Inc., Sanofi K.K., Astellas Pharma Inc.); honoraria (Eisai Co., Ltd, Otsuka Pharmaceutical Co., Ltd, Takeda Pharmaceutical Company Limited., Daiichi Sankyo Company, Limited, Chugai Pharmaceutical Co., Ltd, Novocure, Kowa Company, Ltd). A.N.: consulting fees (NGK Spark Plug); honoraria for lectures (Chugai Pharmaceutical Co., Ltd, Daiichi Sankyo Company, Limited); a board member, unpaid (Japan Society for Neuro-Oncology, Society for Neuro-Oncology). Y.A.: research grants (Eisai Co., Ltd); honoraria (Eisai Co., Ltd). Y.S.: employment (Eisai Co., Ltd).

## Notes

**Authorship statement**. Conception or design of the study: Y.S. Data acquisition: T.I., A.I., Y.H., M.M., K.H., A.N., Y.A., K.I., M.F., and T.K. Data analysis: Y.S. Interpretation of data: T.I., A.I., Y.H., M.M., K.H., A.N., Y.A., K.I., M.F., T.K., and Y.S. Drafting the work or revising it critically for important intellectual content: T.I., A.I., Y.H., M.M., K.H., A.N., Y.A., K.I., M.F., T.K., and Y.S. Final approval of the version to be published: T.I., A.I., Y.H., M.M., K.H., A.N., Y.A., K.I., M.F., T.K., and Y.S. Accountable for all aspects of the work: T.I., A.I., Y.H., M.M., K.H., A.N., Y.A., K.I., M.F., T.K., and Y.S.
